# NTRK3 exhibits a pro‐oncogenic function in upper tract urothelial carcinomas

**DOI:** 10.1002/kjm2.12824

**Published:** 2024-04-09

**Authors:** Lee‐Moay Lim, Yi‐Chen Lee, Ting‐Wei Lin, Zi‐Xuan Hong, Wei‐Chi Hsu, Hung‐Lung Ke, Daw‐Yang Hwang, Wen‐Yu Chung, Wei‐Ming Li, Hui‐Hui Lin, Hung‐Tien Kuo, A‐Mei Huang

**Affiliations:** ^1^ Graduate Institute of Clinical Medicine, College of Medicine Kaohsiung Medical University Kaohsiung Taiwan; ^2^ Division of Nephrology, Department of Internal Medicine Kaohsiung Medical University Hospital, Kaohsiung Medical University Kaohsiung Taiwan; ^3^ School of Medicine, College of Medicine Kaohsiung Medical University Kaohsiung Taiwan; ^4^ Graduate Institute of Medicine, College of Medicine Kaohsiung Medical University Kaohsiung Taiwan; ^5^ Department of Anatomy, School of Medicine, College of Medicine Kaohsiung Medical University Kaohsiung Taiwan; ^6^ Department of Urology, School of Medicine, College of Medicine Kaohsiung Medical University Kaohsiung Taiwan; ^7^ Department of Urology, Kaohsiung Medical University Hospital Kaohsiung Medical University Kaohsiung Taiwan; ^8^ Department of Urology, Kaohsiung Municipal Ta‐Tung Hospital Kaohsiung Medical University Kaohsiung Taiwan; ^9^ National Institute of Cancer Research National Health Research Institute Tainan Taiwan; ^10^ Department of Computer Science and Information Engineering National Kaohsiung University of Science and Technology Kaohsiung Taiwan; ^11^ Department of Urology Ministry of Health and Welfare Pingtung Hospital Pingtung Taiwan; ^12^ Department of Medical Research, Kaohsiung Medical University Hospital Kaohsiung Medical University Kaohsiung Taiwan; ^13^ Doctoral Degree Program in Toxicology, College of Pharmacy Kaohsiung Medical University Kaohsiung Taiwan; ^14^ Department of Biochemistry, School of Medicine, College of Medicine Kaohsiung Medical University Kaohsiung Taiwan; ^15^ Research Center for Environmental Medicine Kaohsiung Medical University Kaohsiung Taiwan

**Keywords:** AKT‐mTOR pathway, neurotrophic receptor tyrosine kinase 3 (NTRK3), prognosis, tropomyosin‐related kinase (TrK), upper tract urothelial carcinoma (UTUC)

## Abstract

Neurotrophic receptor tyrosine kinase 3 (NTRK3) has pleiotropic functions: it acts not only as an oncogene in breast and gastric cancers but also as a dependence receptor in tumor suppressor genes in colon cancer and neuroblastomas. However, the role of NTRK3 in upper tract urothelial carcinoma (UTUC) is not well documented. This study investigated the association between NTRK3 expression and outcomes in UTUC patients and validated the results in tests on UTUC cell lines. A total of 118 UTUC cancer tissue samples were examined to evaluate the expression of NTRK3. Survival curves were generated using Kaplan–Meier estimates, and Cox regression models were used for investigating survival outcomes. Higher NTRK3 expression was correlated with worse progression‐free survival, cancer‐specific survival, and overall survival. Moreover, the results of an Ingenuity Pathway Analysis suggested that NTRK3 may interact with the PI3K‐AKT‐mTOR signaling pathway to promote cancer. NTRK3 downregulation in BFTC909 cells through shRNA reduced cellular migration, invasion, and activity in the AKT‐mTOR pathway. Furthermore, the overexpression of NTRK3 in UM‐UC‐14 cells promoted AKT‐mTOR pathway activity, cellular migration, and cell invasion. From these observations, we concluded that NTRK3 may contribute to aggressive behaviors in UTUC by facilitating cell migration and invasion through its interaction with the AKT‐mTOR pathway and the expression of NTRK3 is a potential predictor of clinical outcomes in cases of UTUC.

## 1 INTRODUCTION

In Taiwan, upper tract urothelial carcinoma (UTUC) is the most common cancer of the renal pelvis and urinary bladder system.^1^ However, UTUC only accounts for 5%–10% of all urothelial carcinoma (UC) cases globally.^2^ UTUC is a public health concern in many countries because 60% of cases are invasive upon diagnosis.^2^ Individuals living in Taiwan are exposed to certain risk factors for UC, including arsenic in drinking water and aristolochic acid in traditional Chinese herbal medications.^3^ The annual incidence rate of UTUC in Taiwan has been estimated at 3.14–3.47 per 100,000 years.^4^ UTUC is unusually common among patients undergoing dialysis (0.9%–1.7%)^5^ and recipients of a kidney transplants (0.1%–1.1%),^6^ with women being overly represented.^4^, ^5^, ^7^ UTUC is associated with a high somatic mutation frequency compared with other solid tumors and has a considerable mutational burden.^8^ UTUC further exhibits considerable histological and molecular intra‐tumoral and inter‐tumoral heterogeneity.^9^

Neurotrophic receptor tyrosine kinase 3 (NTRK3) is a member of the neurotrophin receptor tropomyosin‐related kinase (TrK) family. TrK proteins are tropomyosins fused to a tyrosine kinase domain and are single‐pass transmembrane receptors.^10^ NTRK1, NTRK2, and NTRK3 encode the TrKA, TrKB, and TrKC receptors, which are located on chromosomes 1q23.1, 9q21.33, and 15q25.3, respectively.^11^ TrK receptors and their related neurotrophin ligands exhibit specificity in interactions, regulating survival, growth, differentiation, and apoptosis in the nervous system.^10^ TrKA binds to nerve growth factor, TrKB binds to brain‐derived neurotrophic factor, and neurotrophin‐4/5 while TrKC bind to neurotrophin‐3.^10^

TrKC promotes tumor growth and metastasis, regulates angiogenesis, and prevents apoptosis. Evidence suggests that NTRK3 is not only essential for the development of the nervous system but also plays a critical role in the progression of numerous cancer types, including gastric,^12^ thyroid,^13^ lung,^14^ glial,^15^ and breast cancers.^16^ However, TrKC is not well studied in its role as a dependence receptor (DR) with an oncogenic or tumor‐suppressing function.^17^ DRs trigger two opposing signaling pathways. In the presence of ligands, DRs trigger classic signaling pathways associated with cell survival, migration, and differentiation. In the absence of ligands, DRs provoke apoptosis.^17^ When TrKC is expressed alone, it induces pro‐apoptotic signaling; however, when TrKC binds with its ligand NT‐3, it exhibits an anti‐apoptotic function and acts as an oncogenic factor.^17^ Mutations in NTRK3 promote tumor formation and progression in colorectal cancer.^18^ Zhang et al. identified NTRK3 as a potential prognostic biomarker associated with tumor mutation burden and immune infiltration in bladder cancer after strict screening in a bioinformatics study.^19^ The mechanism and biology of NTRK3 in UTUC remain poor understood. Therefore, the present study investigated the associations among NTRK3 expression, clinicopathological features, and cellular function in UTUC.

## 2 MATERIALS AND METHODS

### 2.1 Clinical samples and clinicopathological data

This study obtained 118 formalin‐fixed UTUC samples from 1997 to 2006 from the Department of Urology, Kaohsiung Medical University Hospital, Taiwan. The Institutional Review Board of Kaohsiung Medical University Hospital approved this study (KMUHIRB‐G(I)‐20150030) and waived the need to obtain written informed consent. The clinical investigators conducted the study according to the principles outlined in the Declaration of Helsinki.

All patients underwent nephroureterectomy and excision of the bladder cuff. Relevant clinical demographic data were collected retrospectively from medical records. Pathologies were classified following the guidelines in the American Joint Committee on Cancer's (AJCC) Cancer Staging Manual.^20^ Patients received regular follow‐up according to the protocol recommended by the National Comprehensive Cancer Network (NCCN). Cystoscopy was performed every 3 months during the first 2 years after surgery, after which cystoscopy was conducted at regular intervals of gradually increasing length, assuming the cancer had not metastasized. Progression‐free survival was calculated from the date of surgery to the date of diagnosis of invasive cancer or distant metastasis. In addition, cancer‐specific survival was calculated from the date of surgery to the date of the patient's death from cancer.

### 2.2 Immunohistochemistry staining

Immunohistochemistry experiments were performed on the obtained samples as previously described.^21^ The processed slides were incubated with a dilution of 1:200 of NTRK3 (TrKC Rabbit mAb [C44H5]#3376, Cell Signaling Technology, Danvers, MA, USA) monoclonal antibodies overnight at 4°C in humidified chambers. The slides were washed three times with a phosphate‐buffered solution, and then incubated with biotinylated secondary antibodies for 30 min at room temperature. The procedures were followed from a previous study^21^ to detect antigen–antibody complexes, perform hematoxylin counterstains, and conduct light microscopy examinations.

### 2.3 Evaluation of immunohistochemical staining

The immunohistochemical staining of the tumor samples was evaluated by two experienced pathologists blinded to any clinical information regarding the patients. After examination, the differences in scoring between pathologists were discussed until a consensus was reached. NTRK3 expression was assessed based on the percentage of positively stained cells and categorized into four quantitative groups: score 1 represents ≤25% positive cells; score 2 corresponds to 26%–50% positive cells; score 3 corresponds to 51%–75% positive cells; and score 4 indicates ≥76% positive cells. In the evaluation of NTRK3 expression, tumors with a score of 1 or 2 were defined as having low expression, and whereas tumors with a score of 3 or 4 were defined as having high expression.

### 2.4 Ingenuity pathway analysis of protein network

The Ingenuity Pathway Analysis (IPA version 68,752,261; QIAGEN Sciences, Germantown, MD, USA) was applied to analyze protein networks in human target genes with NTRK3 as the central molecule. Core analysis was employed to investigate the relationships of NTRK3 and its associated pathways in urothelial cancer as mentioned in our previous publication.^22^ Subsequently, data on the molecules from the QIAGEN knowledge base were integrated into the networks on the basis of the molecules' distinct ontological associations.

### 2.5 Cell lines and culture conditions

UTUC cell lines: UM‐UC‐14 was purchased from the European Collection of Authenticated Cell Cultures in Porton Down, UK and BFTC909 was purchased from Bioresource Collection and Research Center in Hsinchu, Taiwan. Human bladder cancer cell lines: NTUB1 was obtained from National Taiwan University,^23^ and T24 cell line was obtained from the American Type Culture Collection (Manassas, VA, USA). SV40‐immortalized human uroepithelial cell line SV‐HUC1 was obtained from the Bioresource Collection and Research Center and cultured in an F12 medium (Thermo Fisher Scientific, Waltham, MA, USA). The culture conditions were followed the manufacturer's instructions and previous studies.^24^, ^25^

### 2.6 Transfection and clone selection

FLAG‐NTRK3 expression plasmid (HG10048‐CF) was purchased (Sino biological, Inc, Beijing, China). Specific‐NTRK3 targeting shRNA clones were obtained from the RNA Technology Platform and Gene Manipulation Core (National RNAi Core Facility, Academia Sinica, Taipei, Taiwan). The target sequences were as follows: TRCN0000002313: CACGGACATCTCAAGGAATAT; TRCN0000002309: CACTACAACAATGGCAACTAT and TRCN0000194821: CCAATCTACCTGGACATTCTT.

Transfection was performed with using the TurboFect reagent (Thermo Fisher Scientific). The transfected cells were selected by using puromycin (Thermo Fisher Scientific), pooled and designated as shNTRK3(m). The cells were then lysed in a lysis buffer for Western blot analysis. Finally, some of the transfected cells were reseeded onto new plates for further analysis.

### 2.7 Western blot analysis

The treated cells were lysed in a cell extraction buffer (Thermo Fisher Scientific, Hercules, CA, USA), and protein concentration was calculated using the Bio‐Rad Protein Assay Dye Reagent (Bio‐Rad). Equal quantities of protein were electrophoresed on SDS‐PAGE gels and transferred to a PVDF membrane. After blocking with 5% skim milk in Tris‐buffered saline with Tween 20 (TBST), the membranes were incubated with primary antibodies overnight at 4°C. Antibody details are listed in Table S1. The membranes were washed with TBST thrice and incubated with secondary antibodies (anti‐rabbit or anti‐mouse IgG horseradish peroxidase‐conjugated antibodies, 1:5000; Jackson ImmunoResearch West Grove, PA, USA) for 1 h at room temperature. Signals were developed using electro‐chemiluminescence reagents (ECL) (T‐Pro). Finally, the immunoreactive proteins were detected using a MultiGel‐21 gel image system (TOPBIO, New Taipei City, Taiwan) with β‐actin as an internal control.

### 2.8 Wound healing assay

The cells were counted and seeded in a six‐well culture plate after transfection. After incubating the cells overnight, the confluent cell layer was scratched with a pipette tip. The cell gap was then photographed at 0 h and again at 8 h using a phase‐contrast microscope. The healing area was photographed five times at each time point and measured using ImageJ software. The wound closure percentage was expressed as [(area_t=0h_ − area_t=8h_)/area_t=0h_] and comparisons were made against a control group.

### 2.9 Cell invasion assay

Cell invasion ability was measured by QCM ECMatrix cell invasion assay kit (Merck Darmstadt, Germany) followed the procedure of a previous study.^24^

### 2.10 Statistical analysis

SPSS Statistical Package for PC (Version 14.0, SPSS, Chicago, IL, USA) was used for the statistical analyses. A chi‐square test was used to investigate the relationship between NTRK3 expression with various factors, namely gender, age at diagnosis, body mass index, hemodialysis, tumor stage and grade, distant metastasis, and serum creatinine level. Kaplan–Meier estimates were used to generate survival curves, whereas log‐rank tests were used to assess whether the differences between these curves were significant. Hazard ratios and 95% confidence intervals were computed from univariate and multivariate Cox regression models to examine the association between clinicopathological characteristics and survival. Statistical significance was indicated by a *p* value less than 0.05.

## 3 RESULTS

### 3.1 NTRK3 expression in UTUC tissues

**FIGURE 1:**
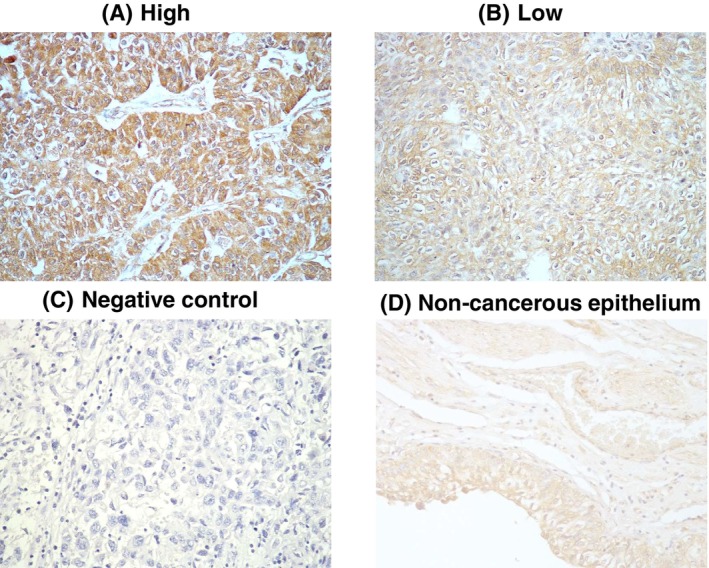
Representative immunohistochemical staining of NTRK3 in upper tract urothelial carcinomas tissues. The level of the NTRK3 expression was divided into (A) high and (B) low as indicated. (C) Negative control and (D) non‐cancerous epithelium were showed. *N* = 118.

*NTRK3* expression in tissue samples from 118 patients with UTUC was analyzed using immunohistochemistry staining techniques. Figure 1 illustrated the staining intensity of NTRK3 in representative tumor samples. The expression of NTRK3 in the samples was classified as high (A), low (B) or negative control (C) according to the level of staining (Figure 1). The non‐cancerous epithelium was displayed in Figure 1D. At least one study found that tumor heterogeneity was associated with poor prognosis and outcomes.^26^ However, low intra‐tumoral heterogeneity was observed in our cohort (data not shown). The baseline characteristics and clinicopathologic variables for our cohort are listed in Table 1.

**TABLE 1:** Correlation of NTRK3 expression with clinicopathological characteristics in patients of upper tract urothelial carcinoma.

Variables	Item	Patient No. (%)	NTRK3				*p* value
			Low		High		
			No.	%	No.	%	
Age (years)		118 (100)	54	45.8	64	54.2	
	<65	38 (32.2)	19	35.2	19	29.7	0.524^a^
	≧65	80 (67.8)	35	64.8	45	67.8	
Gender	Female	66 (55.9)	30	55.6	36	56.2	0.940^a^
	Male	52 (44.1)	24	44.4	28	43.8	
Stage	I/II	77 (65.3)	42	77.8	35	54.7	0.009^a^
	III/IV	41 (34.7)	12	22.2	29	45.3	
Grade	Low	28 (23.7)	15	27.8	13	20.3	0.342^a^
	High	90 (76.3)	39	72.2	51	79.7	
Lymphovascular	Negative	90 (76.3)	47	87	43	67.2	0.012^a^
	Positive	28 (23.7)	7	13	21	32.8	
Creatinine (mg/dL)	≦1.5	64 (54.2)	30	55.6	34	53.1	0.792^a^
	>1.5	54 (45.8)	24	44.4	30	46.9	
Bladder recurrence	Negative	85 (72.0)	40	74.1	45	70.3	0.650^a^
	Positive	33 (28.0)	14	25.9	19	29.7	
Cancer progression survival	Negative	98 (83.1)	51	94.4	47	73.4	0.003^b^
	Positive	20 (16.9)	3	5.6	17	26.6	
Cancer death	No	100 (84.7)	52	96.3	48	75	0.002^b^
	Yes	18 (15.3)	2	3.7	16	25	

^a^ The *p* value was calculated by the chi‐square test.

^b^ The *p* value was calculated by the Fisher's exact test.

Sixty‐four (54.2%) of the cancer samples exhibited high levels of NTRK3 expression. Significant correlations were observed between NTRK3 expression and cancer stage (*p* = 0.009), lymphovascular invasion (*p* = 0.012), cancer progression (*p* = 0.003), and cancer death (*p* = 0.002) (Table 1). By contrast, no correlation was observed between NTRK3 expression and age, gender, tumor grade, serum creatinine, or bladder recurrence.

Table 2 details the univariate and multivariate analysis results for UTUC progression‐free survival (Table 2A), cancer‐specific survival (Table 2B) and overall survival (Table 2C). Tumor stage, lymphovascular invasion, and NTRK3 expression were significant factors in the univariate analysis of all outcomes. However, only the tumor stage remained significantly associated with all outcomes in the multivariate analysis.

**TABLE 2A:** Univariate and multivariable analysis of progression‐free survival for upper tract urothelial carcinoma.

Variables	Item	Univariate			^a^Multivariable		
		HR	95% CI	*p* value	HR	95% CI	*p* value
Age (years)	≧65	0.94	(0.36, 2.46)	0.900	‐	‐	‐
	< 65	1.00		‐			
Gender	Male	1.23	(0.51, 2.95)	0.650	‐	‐	‐
	Female	1.00		‐			
Stage	III/IV	13.8	(4.03, 47.22)	<0.001	10	(2.76, 36.27)	<0.001
	I/II	1.00			1.00		
Grade	High	1.53	(0.51, 4.63)	0.448	‐	‐	‐
	Low	1.00		‐			
Lymphovascular	Positive	3.31	(1.36, 8.03)	0.008	1.15	(0.45, 2.91)	0.774
	Negative	1.00			1.00		
Creatinine (mg/dL)	>1.5	0.64	(0.26, 1.60)	0.340	‐	‐	‐
	≦1.5	1.00		‐			
NTRK3	High	5.79	(1.69, 19.80)	0.005	3.13	(0.88, 11.18)	0.078
	Low	1.00			1.00		

^a^ Variables with *p* < 0.05 on univariate analysis were included in multivariable analysis.

**TABLE 2B:** Univariate and multivariable analysis of cancer‐specific survival for upper tract urothelial carcinoma.

Variables	Item	Univariate			^a^Multivariable		
		HR	95% CI	*p* value	HR	95% CI	*p* value
Age (years)	≧65	1.14	(0.40, 3.19)	0.809	‐	‐	‐
	<65	1.00		‐			
Gender	Male	1.26	(0.50, 3.17)	0.627	‐	‐	‐
	Female	1.00		‐			
Stage	III/IV	39.8	(5.27, 300.62)	<0.001	27.2	(3.46, 214.10)	0.002
	I/II	1.00			1.00		
Grade	High	1.83	(0.53, 6.37)	0.342	‐	‐	‐
	Low	1.00		‐			
Lymphovascular	Positive	3.79	(1.50, 9.58)	0.005	1.21	(0.47, 3.15)	0.691
	Negative	1.00			1.00		
Creatinine (mg/dL)	>1.5	1.00	(0.40, 2.54)	0.995	‐	‐	‐
	≦1.5	1.00		‐			
NTRK3	High	7.9	(1.81, 34.42)	0.006	3.65	(0.82, 16.37)	0.091
	Low	1.00			1.00		

^a^ Variables with *p* < 0.05 on univariate analysis were included in multivariable analysis.

**TABLE 2C:** Univariate and multivariable analysis of overall survival for upper tract urothelial carcinoma.

Variables	Item	Univariate			^a^Multivariable		
		HR	95% CI	*p* value	HR	95% CI	*p* value
Age (years)	≧65	1.19	(0.50, 2.84)	0.691	‐	‐	‐
	<65	1.00		‐			
Gender	Male	0.93	(0.43, 2.02)	0.848	‐	‐	‐
	Female	1.00		‐			
Stage	III/IV	9.51	(3.55, 25.51)	<0.001	7.62	(2.69, 21.59)	<0.001
	I/II	1.00			1.00		
Grade	High	1.73	(0.64, 4.69)	0.281	‐	‐	‐
	Low	1.00		‐			
Lymphovascular	Positive	2.58	(1.15, 5.77)	0.021	1.04	(0.44, 2.43)	0.933
	Negative	1.00			1.00		
Creatinine (mg/dL)	>1.5	0.96	(0.44, 2.10)	0.915	‐	‐	‐
	≦1.5	1.00		‐			
NTRK3	High	3.94	(1.48, 10.48)	0.006	2.39	(0.87, 6.53)	0.091
	Low	1.00			1.00		

^a^ Variables with *p* < 0.05 on univariate analysis were included in multivariable analysis.

**FIGURE 2:**
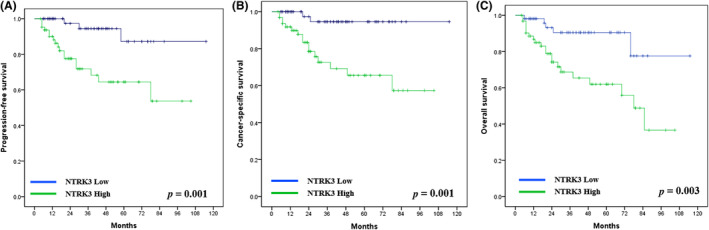
The Kaplan–Meier curves for UTUC (A) progression‐free survival, (B) cancer‐specific survival and (C) overall survival. The Kaplan–Meier analysis for clinical outcomes according to NTRK3 immunoexpression status. The numbers of clinical samples in each group were shown.

The Kaplan–Meier curves for UTUC progression‐free survival and cancer‐specific survival are illustrated in Figure 2. As Figure 2A–C indicate, patients with high NTRK3 expression had worse progression‐free survival (*p* = 0.001), cancer‐specific survival (*p* = 0.001) and overall survival (*p* = 0.003).

### 3.2 Exploration of *NTRK3* ‐related cell signaling pathway

**FIGURE 3:**
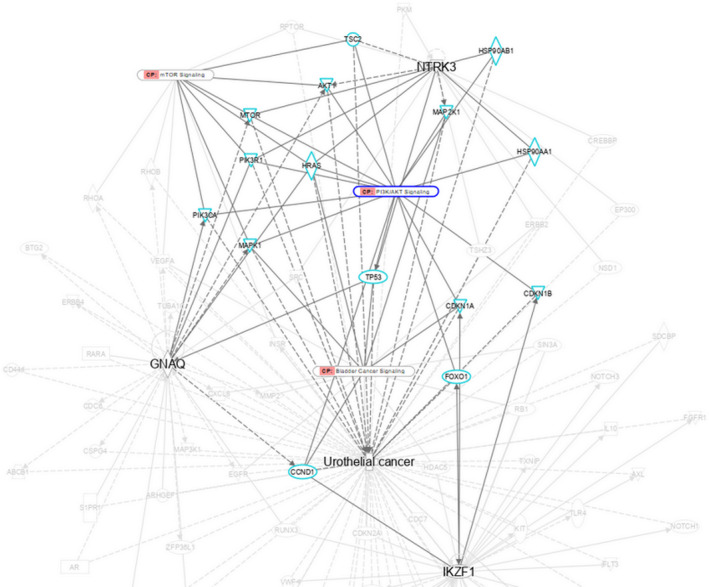
NTRK3 signal transduction pathways analysis by IPA. Protein network of urothelial cancer (UC) relevant target proteins with canonical pathway (CP) was show. NTRK3 was represented in bold as focus molecule which related to UC. The solid line represents a direct interaction between the two genes whereas the dashed line means there is an indirect association to proteins, pathways and disease. The label with CP tag means canonical pathway. The molecules associated with PI3K/AKT signaling canonical pathway in first level were highlighted and the second level ones were faded out. The molecules label in cyan were genes associated to NTRK3.

IPA canonical pathway analysis was used to explore the signal transduction pathways of NTRK3 and the implications of these pathways for clinical application. As shown in Figure 3, NTRK3 exhibited promising interactions with the phosphatidylinositol 3‐kinase (PI3K)‐AKT‐mTOR signaling pathway. Therefore, the PI3K‐AKT‐mTOR signaling pathway will be further investigated.

### 3.3 NTRK3 expression in UTUC cell lines

**FIGURE 4:**
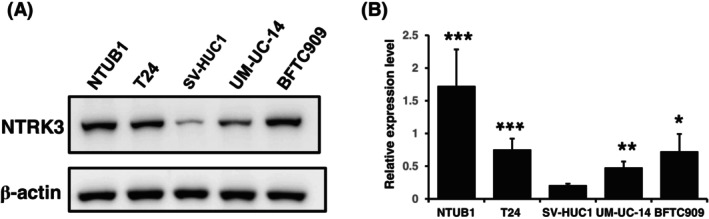
NTRK3 protein expression is higher in UC cell lines. (A) NTRK3 protein expression was examined by Western blot analysis in SV‐40 immortalized human urothelial cells (SV‐HUC1), two UCB cell lines (NTUB1 and T24) and two UTUC cell lines (UM‐UC‐14 and BFTC909). Beta‐actin was used as a loading control. (B) The quantification of the data. *N* = 5. *p < 0.05; **p < 0.01, *p* < *** 0.001.

NTRK3 protein expression was examined in UC cell lines and compared with SV40‐immortalized human uroepithelial cells SV‐HUC1 in a Western blot analysis. As shown in Figure 4, NTRK3 protein expression was strongly associated with the level of UTUC aggressiveness (in order from most to least aggressive: BFTC909 > UM‐UC‐14 > SV‐HUC1). The UC bladder (UCB) cell lines (NTUB1 and T24) had significantly higher *NTRK3* protein expression than the SV‐HUC1 cells.

### 3.4 NTRK3 expression associated with cellular migration and invasion

**FIGURE 5:**
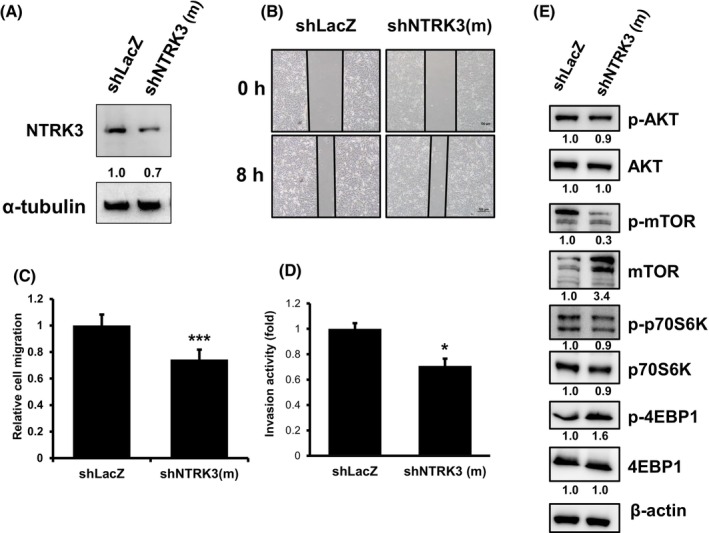
Knocking‐down *NTRK3* suppresses cellular migration and invasion activity in BFTC909 cells. (A) The NTRK3 expression was checked by Western blot analysis in NTRK3 shRNAs (shNTRK3(m)) and negative control shLacZ BFTC909 cells. α‐tubulin was used as a loading control. (B) The cells were the subjected for cellular migration assay and the quantification of migration was shown in (C), *N* = 4. (D) Cell invasion ability was analysis by QCM ECMatrix cell invasion assay, *N* = 7. * *p* < 0.05; *p* < *** 0.001, respectively. (E) the AKT–mTOR pathway proteins were examined, respectively. Beta‐actin was used as a loading control.

BFTC909 and UM‐UC‐14 cell lines were employed to investigate the role of NTRK3. A knocked‐down *NTRK3* shRNA stable pooled clone (shNTRK3(m)) was successfully generated in an NTRK3‐relative high cell line (Figure 4A), BFTC909. NTRK3 protein expression was reduced by approximately 30% compared with the negative control shLacZ cells (Figure 5A). In addition, shNTRK3(m) suppressed cell migration (Figure 5B,C) and reduced invasiveness (Figure 5D) compared with the shLacZ cells. The AKT‐mTOR‐p70S6K‐4EBP1 axis was also noticeably affected by the knocked‐down NTRK3 expression (Figure 5E).

**FIGURE 6:**
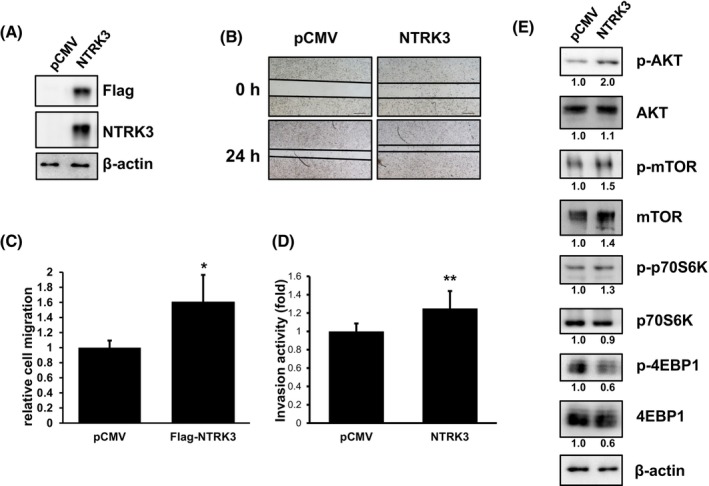
*NTRK3* overexpression enhanced cellular migration and invasion activity in UM‐UC‐14 cells. (A) The NTRK3 expression was checked by Western blot analysis in pCMV and FLAG‐NTRK3 UM‐UC‐14 cells. β‐actin was used as a loading control. (B) The cells were the subjected for cellular migration assay and the quantification of migration was shown in (C), *N* = 4. (D) Cell invasion ability was analysis by QCM ECMatrix cell invasion assay, *N* = 4. *, *p* < 0.05; **, *p* < 0.01, respectively. (E) The AKT‐mTOR pathway proteins were examined, respectively. Beta‐actin was used as a loading control.

We then next over‐expressed FLAG‐tagged NTRK3 using transiently transfection in a UM‐UC‐14 cell line with a relatively low endogenous NTRK3 level (Figure 4A). As shown in Figure 6, NTRK3 exhibited increased cell migration, invasiveness, and activation of AKT‐mTOR‐p70S6K‐4EBP1 pathway compared with the vector pCMV control.

On the basis of these findings, we conclude that NTRK3 play an oncogenic role in UTUC cells. The underlying molecular mechanism of this role will be investigated in a future study.

## 4 DISCUSSION

NTRK3 expression was associated with worse progression‐free survival, cancer‐specific survival, and overall survival in UTUC tissues. In addition, NTRK3 may mediate cell migration and invasiveness by interacting with AKT‐mTOR signaling pathways in UTUC. This study is the first to evaluate the associations among NTRK3 expression, clinicopathological features, and cellular function in UTUC.

UTUC is a cancer subgroup originating in the urothelium of the renal pelvis or ureter that accounts for 5%–10% of all UC cases.^2^ UTUC has a particularly aggressive disease phenotype, with invasion observable at diagnosis in approximately 60% of UTUC case.^2^ The mean age at diagnosis is approximately 73 years, and men are overrepresented at a ratio of between 2 and 3 to 1. The estimated worldwide annual incidence is two cases per 100,000 people.^27^ However, UTUC in Taiwan is estimated to account for 30%–40% of all UC cases, a rate far higher than the global incidence.^28^

The increased prevalence of UTUC in Taiwan may be due to the presence of carcinogens such as aristolochic acid and cyclophosphamide in traditional Chinese medical treatments or to environmental factors that contribute to tumor development such as high rates of smoking and vasculitis.^3^, ^29^ Our previous study demonstrated that UC was most common renal pelvic or bladder malignancy observed following kidney transplants in Taiwan.^30^ Increasing evidence from genomic studies has gradually revealed the molecular landscape of UTUC and identifying numerous gene alterations that are promising targets for gene therapies.

The mutated oncogenes or tumor suppressors discovered in UTUC include FGFR3, TP53, and the chromatin remodelers KMT2D and KDM6A.^8^ FGFR3 mutations were detected in more than 90% of low‐grade tumors, whereas TP53 mutations were only observed in high‐grade tumors.^8^ Additionally, mutations in KMT2D and KDM6A were detected in both low‐grade and high‐grade UTUC.^8^ However, NTRK3 mutation is less commonly reported in cases of UTUC. For example, Sfakianos et al. documented NTRK3 gene alteration in just 4.71% of cases of UTUC in their next‐generation targeted sequencing of 300 cancer‐associated genes.^31^ In another study on the genomic differences between metastatic lower tract UC and metastatic upper tract UC, NTRK3 mutations were detected in metastatic lower tract UC using cell‐free circulating DNA next‐generation sequencing.^32^ Furthermore, in our previous study, we found that NTRK3 mutation was a unique feature in the UC tissue of recipients of kidney transplants.^22^ Another study also suggested that NTRK3 was a potential prognostic biomarker associated with tumor mutation burden and immune infiltration in bladder cancer.^19^

NTRK3 encodes the TrKC protein, a member of the TrK family. After binding to the ligand NT3 receptor, TrKC auto‐phosphorylates and activates various intracellular signaling pathways, comprising JAK/STAT, PI3K/AKT, phospholipase C γ, and Ras/MEK/ERK, to promote proliferation, differentiation, and survival.^10^ These pathways mediate the effects of neurotrophin on neural cell proliferation, neuronal migration, and differentiation.^10^ For this reason, TrK aberrations are responsible for gene fusions, gene overexpression, and single‐nucleotide variations in the pathogenesis of many cancer.^33^ NTRK3 has been classified as an oncogene in breast cancer and gastric cancer.^12^, ^16^ However, it acts as a tumor suppressor gene in colon cancer and neuroblastomas.^18^, ^34^ Moreover, NTRK3 plays a pleiotropic role in regulating the pathobiology of many cancers. Sasahira et al. examined NTRK3 as a candidate gene for accelerated angiogenesis and lymphangiogenesis in oral squamous cell carcinoma and observed a high expression of NTRK3 in cases with nodal metastasis and poor prognosis.^35^ In addition to the positive associations between NTRK3 expression and cancer stage, lymphovascular invasion, cancer progression, and cancer death, our findings suggested that NTRK3 may exert aggressive behaviors in UTUC by mediating cell migration and invasiveness through its interaction with the AKT‐mTOR signaling pathway. This hypothesis remains to be tested in future studies.

NTRK3 promotes survival and neurogenesis through the PI3K and MEK pathways, which are common downstream substrates of many receptor tyrosine kinases.^36^ The PI3K/AKT pathway is crucial in cancer cases because it is a key regulator of cell survival under stress condition.^36^ At least one study has reported that changes in protein expression along the PI3K/AKT pathway are associated with UTUC aggressiveness.^37^ The dysregulation of the PI3K/AKT pathway in breast cancer caused by the overexpression of NTRK3 was also reported in another study by Jin et al.^16^ The effects of changes in the NTRK3 protein on the activity of the PI3K‐AKT pathway have not been well studied in cases of UTUC. In the results of IPA analysis in this study, NTRK3 appeared to affect the AKT–mTOR signaling pathway. Huan, J, et al. reported that AKT/mTOR modulated the migration and invasion activity and played an important role in the progression of bladder cancer^38^ which is consistent with our findings (Figures 5 and 6). The role of NTRK3 in the PI3K/AKT‐mTOR downstream pathway in UTUC tumorigenesis requires further investigation.

## 5 CONCLUSION

**FIGURE 7:**
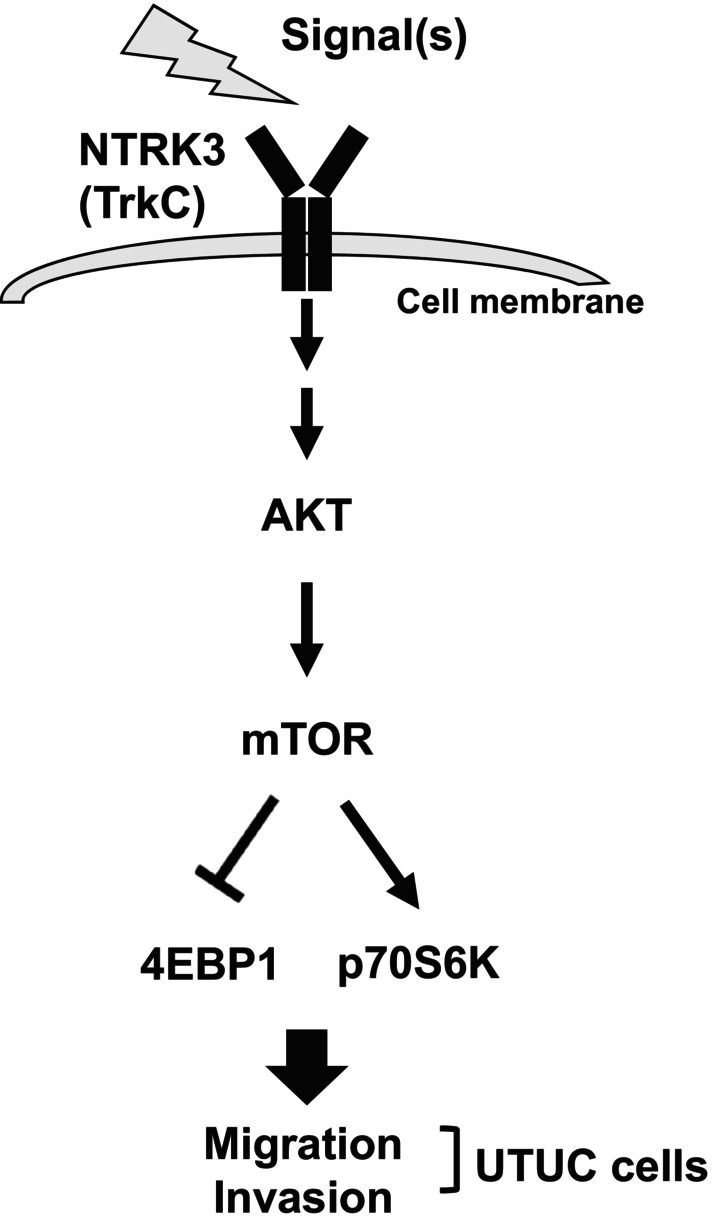
NTRK3 enhances cancer cell migration and invasion by promoting AKT–mTOR pathway. The diagram showed a hypothesized model to summarize the signaling pathways mediated by NTRK3 to promote cellular migration and invasion activity in UTUC.

Higher NTRK3 expression was associated with worse progression‐free survival, cancer‐specific survival and overall survival in patients with UTUC in this study. The expression of NTRK3 promoted cellular migration and invasion in UTUC cells. The results of our analysis revealed that NTRK3 affects the AKT‐mTOR‐p70S6K‐4EBP1 signaling pathways and has the phenotypes depicted in Figure 7. These results indicate that NTRK3 is a promising prognostic biomarker and a valuable target in treatments for UTUC.

## CONFLICT OF INTEREST STATEMENT

The authors have no conflicts of interest to declare.

## Supporting information


**Table S1.** The antibodies used in this study are listed.
