# Supplementary material for: NTRK3 exhibits a pro‐oncogenic function in upper tract urothelial carcinomas
Source: Kaohsiung J Med Sci. 2024 Apr 9;40(5):445–55. doi: 10.1002/kjm2.12824 (PMC11895571; doi:10.1002/kjm2.12824)
Supplement: Supplementary file 1 — Table S1. The antibodies used in this study are listed. [file KJM2-40-445-s001.docx]

Supplementary Table S1

| **Antibodies for** | **SOURCE** | **Cat. No.** |
| --- | --- | --- |
| NTRK3 | Cell Signaling Technology | 3376 |
| p-mTOR | Cell Signaling Technology | 5536 |
| mTOR | Cell Signaling Technology | 2983 |
| p-p70S6K | Cell Signaling Technology | 9234 |
| p70S6K | Cell Signaling Technology | 2708 |
| p-AKT | Cell Signaling Technology | 9271 |
| AKT | Proteintech | Pro-10176 |
| p-4EBP1 | Cell Signaling Technology | 2855 |
| 4EBP1 | Cell Signaling Technology | 9644 |
| β-actin | BD Transduction Laboratories | 612656 |

The antibodies used in this study are listed.
